# Supplementary material for: An HDAC6-dependent surveillance mechanism suppresses tau-mediated neurodegeneration and cognitive decline
Source: Nat Commun. 2020 Nov 2;11:5522. doi: 10.1038/s41467-020-19317-4 (PMC7606452; doi:10.1038/s41467-020-19317-4)
Supplement: Supplementary file 1 — Supplementary Information [file 41467_2020_19317_MOESM1_ESM.pdf]

Supplementary Information

An HDAC6-dependent surveillance mechanism suppresses tau-mediated neurodegeneration and cognitive decline

Trzeciakiewicz et al.

This file contains:

Supplementary Figures 1-10

Supplementary Tables 1-2

Supplementary Figures

Supplementary Fig. 1

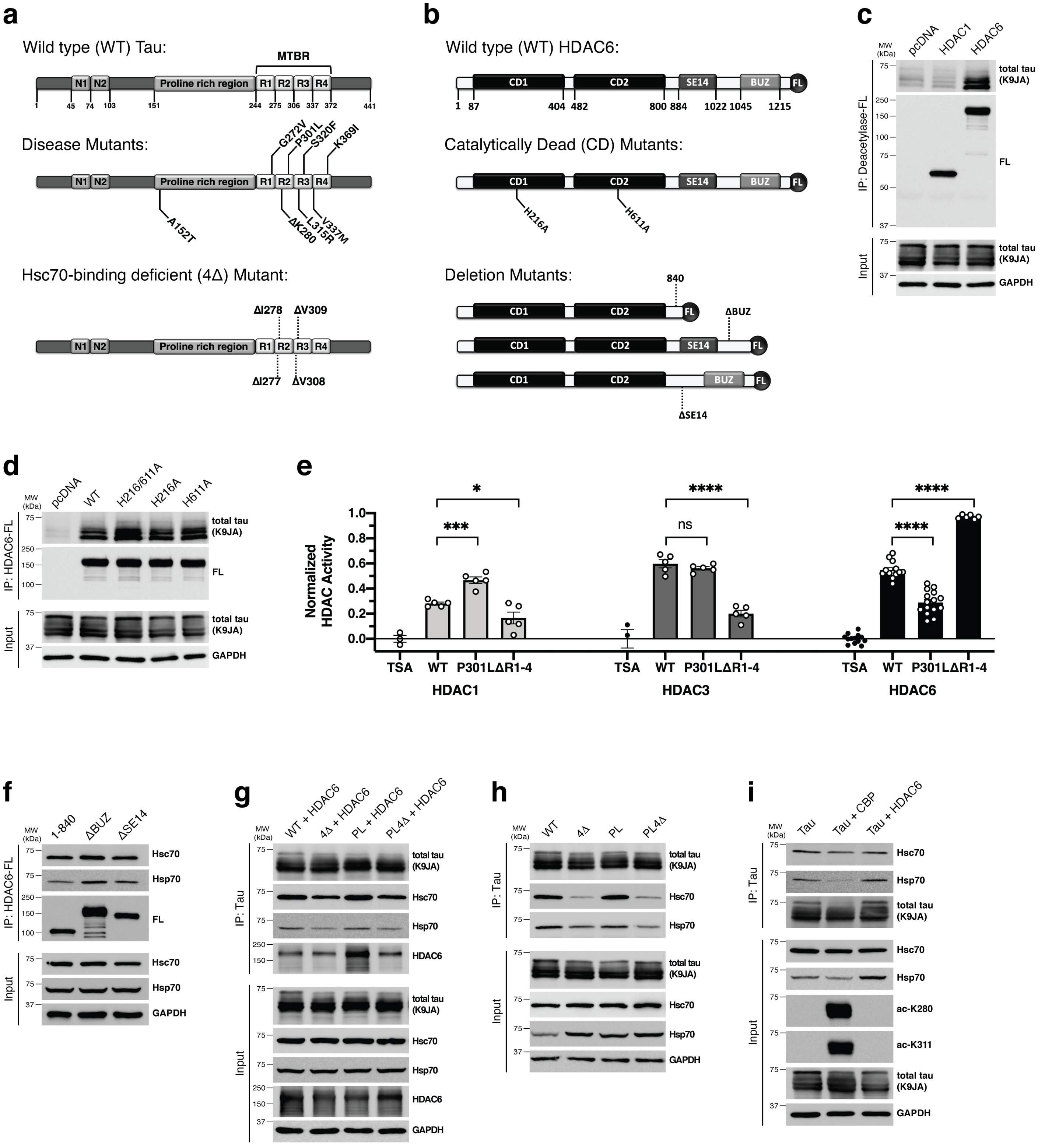

Supplementary Fig. 1 Tau-chaperone interactions dictate HDAC6 binding. **a** Schematic of tau constructs used in this study: wild type (WT) human full-length tau-T40 (2N4R-tau), FTD-disease linked mutants, and Hsc/Hsp70-binding deficient (4Δ) mutants. **b** Schematic of FLAG (FL)-tagged HDAC6 constructs used in this study: WT human full-length HDAC6, Catalytically Dead (CD) mutants either containing H216A or H611A single mutations or both H216/611A mutations, 1-840 (lacking C-terminus 841-1215), ΔBUZ (lacking polyubiquitin binding domain 1045-1215), and ΔSE14 (lacking the SE14 domain mediating protein-protein interactions 884-1022). **c** Co-IP assay was performed to evaluate tau binding to mock pcDNA, FL-tagged HDAC1, or FL-tagged HDAC6. **d** Co-IP assay was performed to evaluate tau binding to mock pcDNA, FL-tagged WT HDAC6, or Catalytically Dead HDAC6 mutants containing both H216/611A mutations or single H216A or H611A. **e** HDAC1, HDAC3, and HDAC6 activity assays were used to evaluate the ability of full-length recombinant WT, P301L, and ΔR1-4 tau proteins to inhibit HDAC activity, which were normalized to the TSA control. **f** Co-IP assay was performed to evaluate which HDAC6 domain binds endogenous Hsc/Hsp70. HDAC6 1- 840 and ΔSE14 showed decreased binding to both Hsc70 and Hsp70. **g** Co-IP assay was performed to evaluate tau binding to WT HDAC6 and Hsc/Hsp70 via tau pull-down (compared to Fig. 1l with HDAC6 pull-down). The Hsc/Hsp70 binding-deficient tau mutants 4Δ and PL4Δ (deletion of residues I277/I278/I308/V309) showed decreased binding to HDAC6 and heat shock proteins. **h** Co-IP confirming Hsc/Hsp70 deficient tau mutant proteins (4Δ and PL4Δ) are deficient in binding endogenous Hsc/Hsp70. **i** Co-IP assay to evaluate whether tau acetylation alters binding to endogenous Hsc/Hsp70. Acetylated tau was generated by co-transfection with CBP, resulting in decreased binding to Hsps (see middle lane). Fully deacetylated tau was generated by co-transfection with HDAC6, which resulted in slightly increased tau binding to Hsps. Statistical tests: p value determined by two-sided unpaired t-test from n = 3 biologically independent experiments (e). Representative co-IP assays and immunoblotting data are shown from n = 3 (e), n = 2 (c, d, f) and n = 1 (g-i) biologically independent experiments. Error bars represent ± SEM. *$p < 0.05$; ***$p < 0.001$; ****$p < 0.0001$. Source data are provided as a Source Data file.

Supplementary Fig. 2

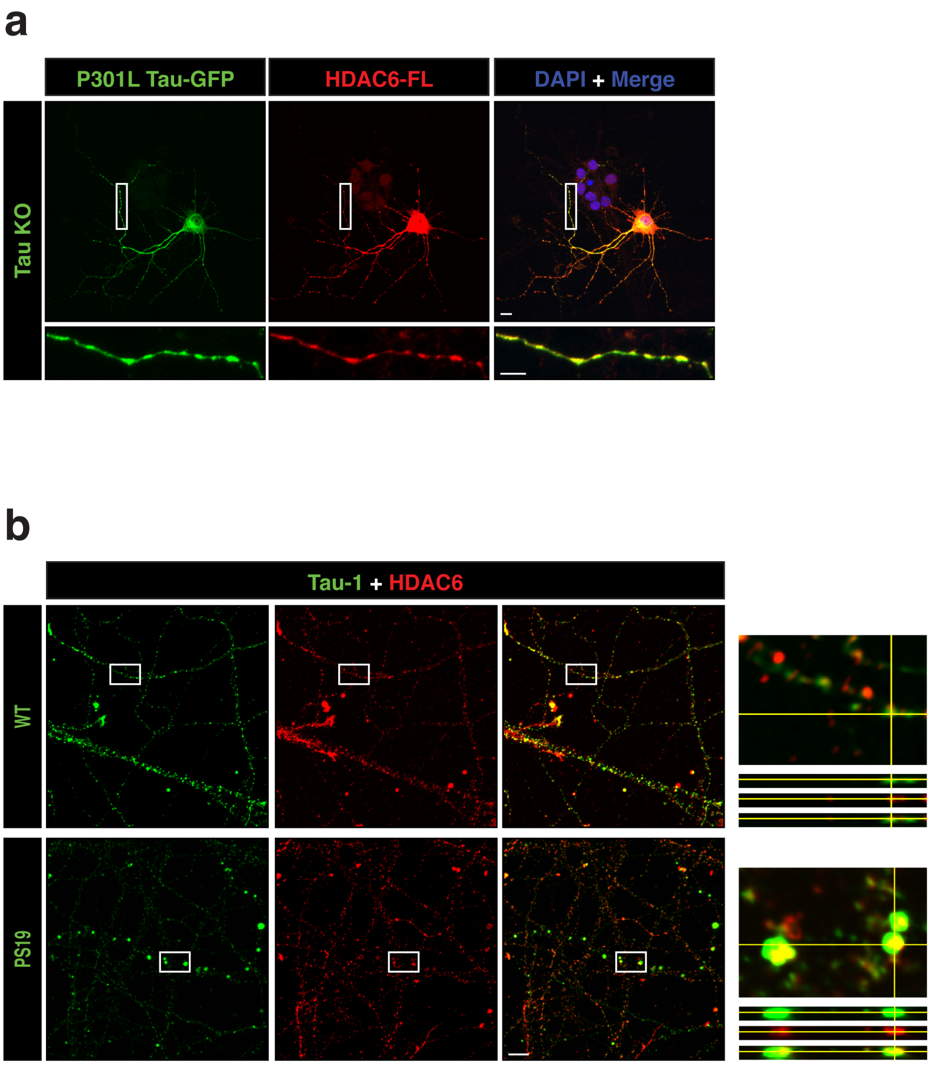

Supplementary Fig. 2 Co-localization of tau and HDAC6 in primary cultured neurons by confocal microscopy. **a** Primary cortical neurons derived from embryos (E16) tau knockout (KO) mice were cultured for 10 days in vitro (10 DIV) and transfected with P301L Tau-GFP and HDAC6-FL expression constructs followed by confocal imaging. Top row, scale bar = 10 µm. Bottom row, scale bar = 5 µm. **b** Primary cortical neuron cultures (E16) from WT (top) and PS19 tau transgenic (bottom) mice showed co-localized focal beading (or swellings) of tau (green) and endogenous HDAC6 (red). The small white rectangle highlights a magnified inset of a neuronal process harboring tau-positive foci. Scale bar = 10 µm. Orthogonal perspective (derived from ImageJ) of co-localized foci were provided for both WT and PS19 neurons. Representative confocal images are shown from n = 3 biologically independent experiments (a, b).

Supplementary Fig. 3

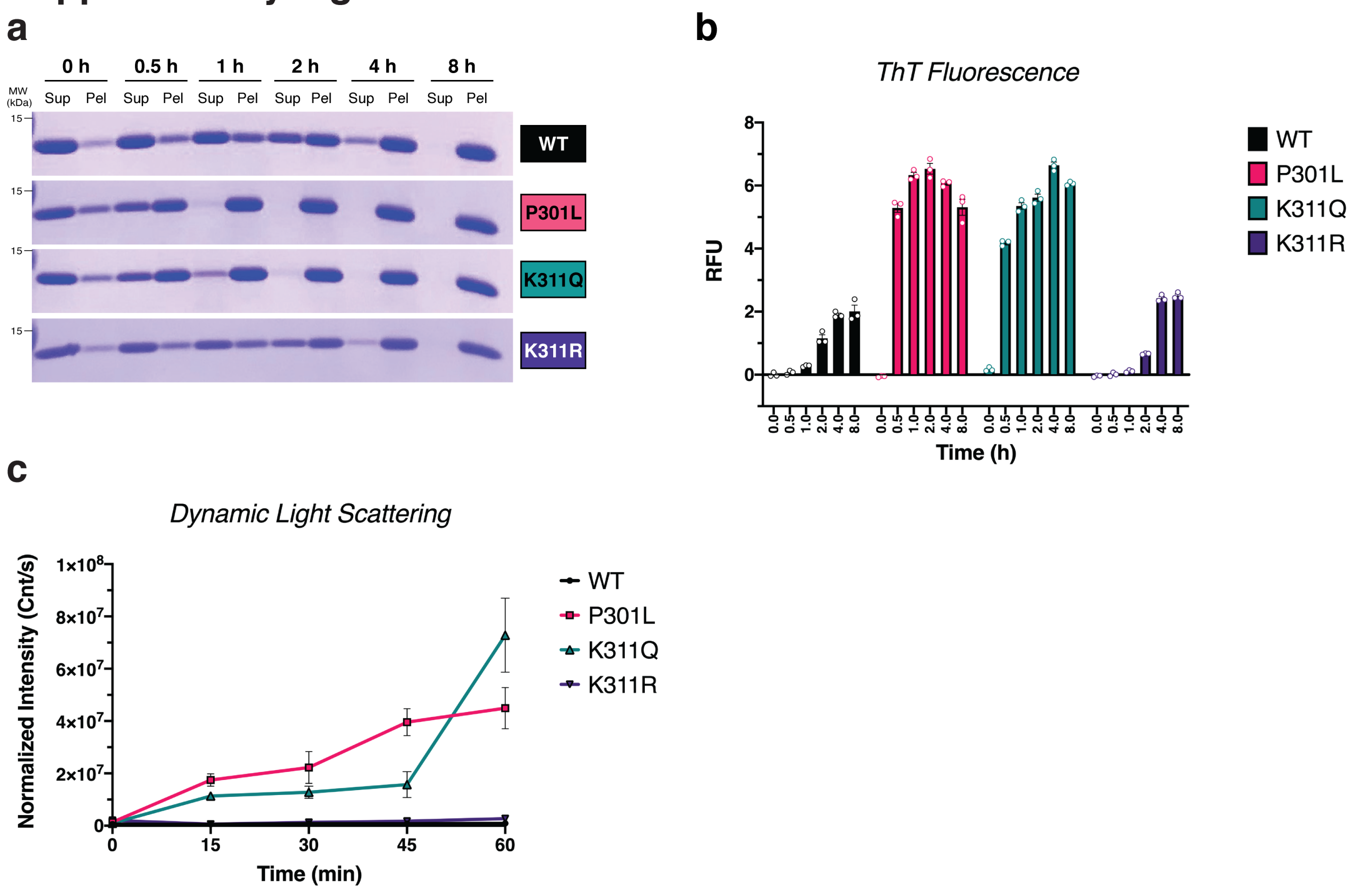

Supplementary Fig. 3 The K311Q acetylation mimic mutant displays enhanced tau aggregation propensity comparable to the disease linked P301L mutant. **a** Purified recombinant tau K18 WT, P301L, K311Q, and K311R tau proteins (10 μM) were fibrillized using heparin as an inducing agent and soluble supernatant (Sup) and insoluble pellet (Pel) fractions were separated by centrifugation followed by Coomassie blue staining. Fractions were analyzed at the indicated time points from 0 – 8 hr post fibrillization. A representative Coomassie stained gel is shown from n = 2 biologically independent experiments. **b** Thioflavin T (ThT) fluorescence of purified K18 tau protein fibril reactions incubated with heparin for indicated time points. Values represent means ± SEM from n = 6 independent biological replicates. **c** Dynamic light scattering normalized intensity measurements of purified tau K18 protein fibril reactions (WT, P301L, K311Q, and K311R) from the indicated time points after induction with heparin. Values represent means ± SEM from n = 10 individual biological replicates. Source data are provided as a Source Data file.

Supplementary Fig. 4

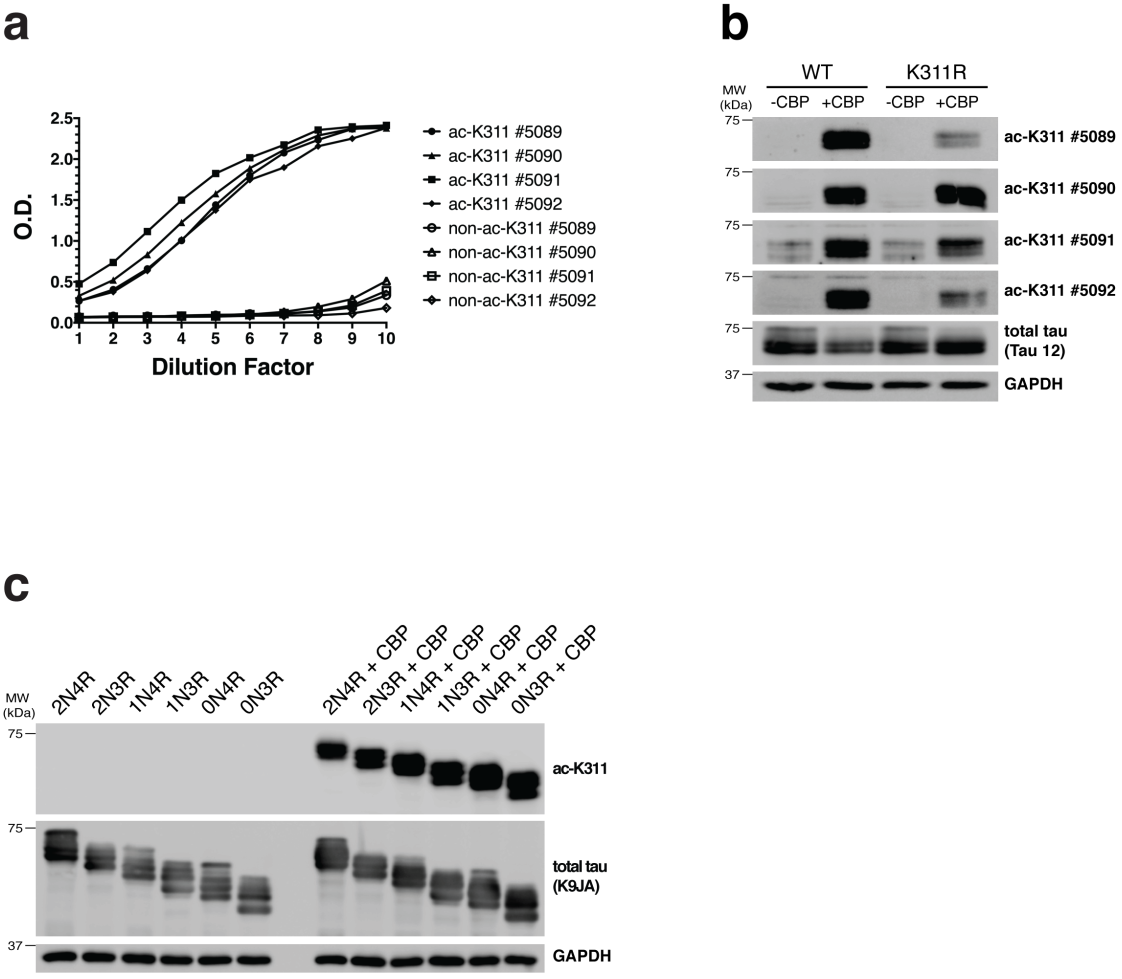

Supplementary Fig. 4 Validation of acetylation-specificity and lysine site-specificity of polyclonal ac-K311 tau antibodies. **a** ELISA results of four polyclonal ac-K311 antibodies (#5089, #5090, #5091, #5092) generated by rabbit immunization (Genscript, Piscataway, NJ). Indirect ELISA assays were performed with acetylated peptide (^{307}QIVYK[Ac]PVDLSKVTSC^{320}) or non-acetylated peptide (^{307}QIVYKPVDLSKVTSC^{320}) as coating antigens used at 4.0 µg/ml (100 µl/well) in coating buffer (Phosphate Buffered Saline, pH 7.4). Primary antibodies were detected with peroxidase-conjugated secondary antibodies (goat anti-rabbit IgG). **b** Immunoblotting analysis of WT tau and the non-acetylatable K311R mutant in the absence (mock empty pcDNA3.1 vector, -CBP) or presence of CBP acetyltransferase (+CBP). Acetylation-specific ac-K311 antibodies are expected to show low/minimal immunoreactivity in the presence of the K311R mutant to demonstrate lysine site-specificity. **c** Immunoblotting analysis of the six tau isoforms in the absence (mock empty pcDNA3.1 vector) or presence of CBP acetyltransferase. The tau isoforms vary in the number of N-terminal repeats (0N, 1N, or 2N) and MTBR domains (3R or 4R). The polyclonal ac-K311 antibody recognizes acetylated residue K311 within R3 of the tau MTBR and detected all acetylated tau isoforms when HEK-293A cells were co-transfected with CBP in vitro. Representative immunoblotting is shown from n = 2 (b, c) biologically independent experiments. Source data are provided as a Source Data file.

Supplementary Fig. 5

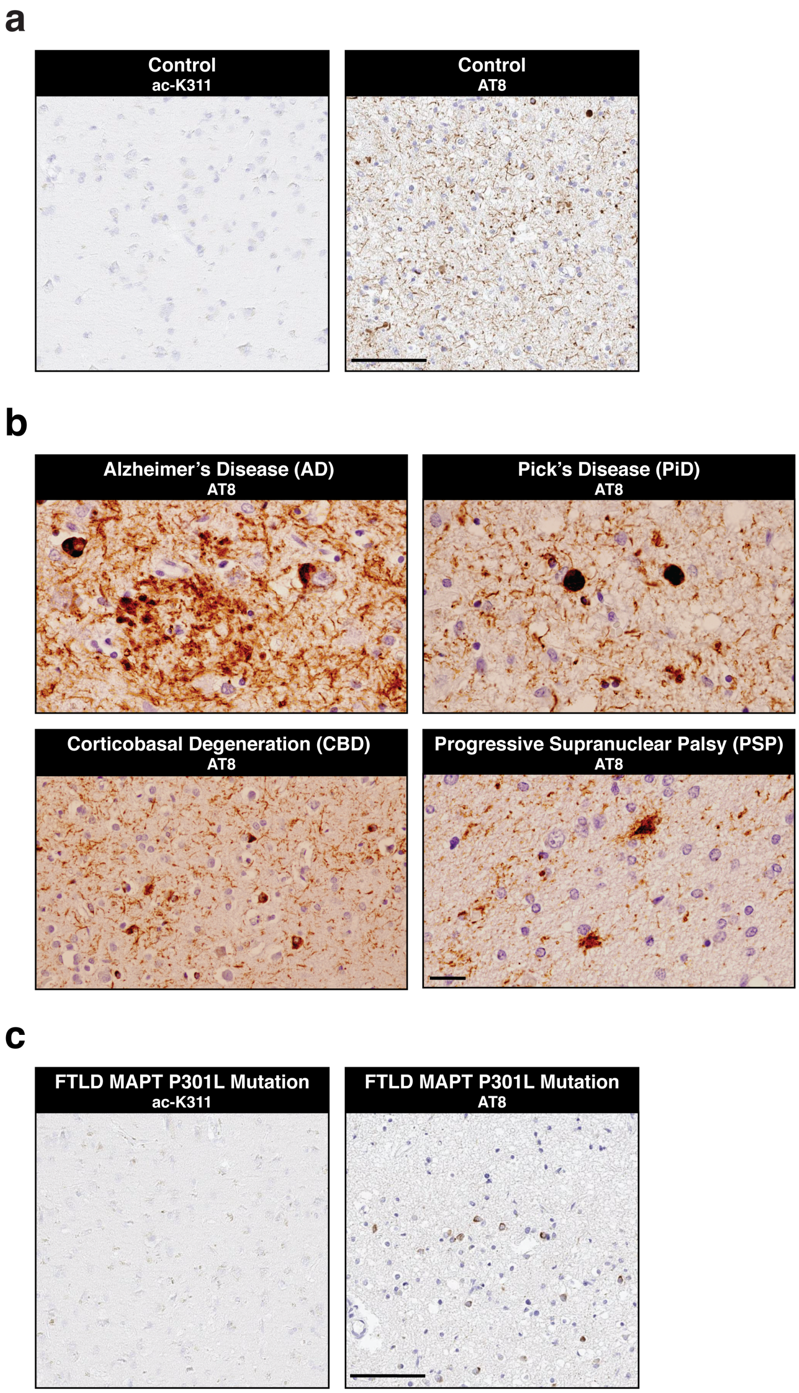

Supplementary Fig. 5 Characterization of positive and negative controls for tau acetylation and tau phosphorylation by IHC analysis of tauopathy cases. **a** IHC analysis of control brain using ac-K311 and AT8 antibodies. Scale bar = 100 µm. **b** Phosphorylated tau inclusions were examined by IHC analysis using the AT8 (p-S202/T205) antibody in 3R, 3R/4R, and 4R tauopathies: Alzheimer's disease (AD), Pick's disease (PiD), corticobasal degeneration (CBD), and progressive supranuclear palsy (PSP). All tauopathies demonstrated AT8-positive pathological lesions in contrast to ac-K311, which showed specificity for mixed 3R/4R (AD) and 3R (PiD) tauopathy cases (Fig. 3c). Scale bar = 100 µm. **c** IHC analysis of an FTLD MAPT P301L human case using ac-K311 and AT8 antibodies. Scale bar = 100 µm. Representative bright-field images are shown from n = 5 (a), n = 3 (b), and n = 2 (c) independent experiments.

Supplementary Fig. 6

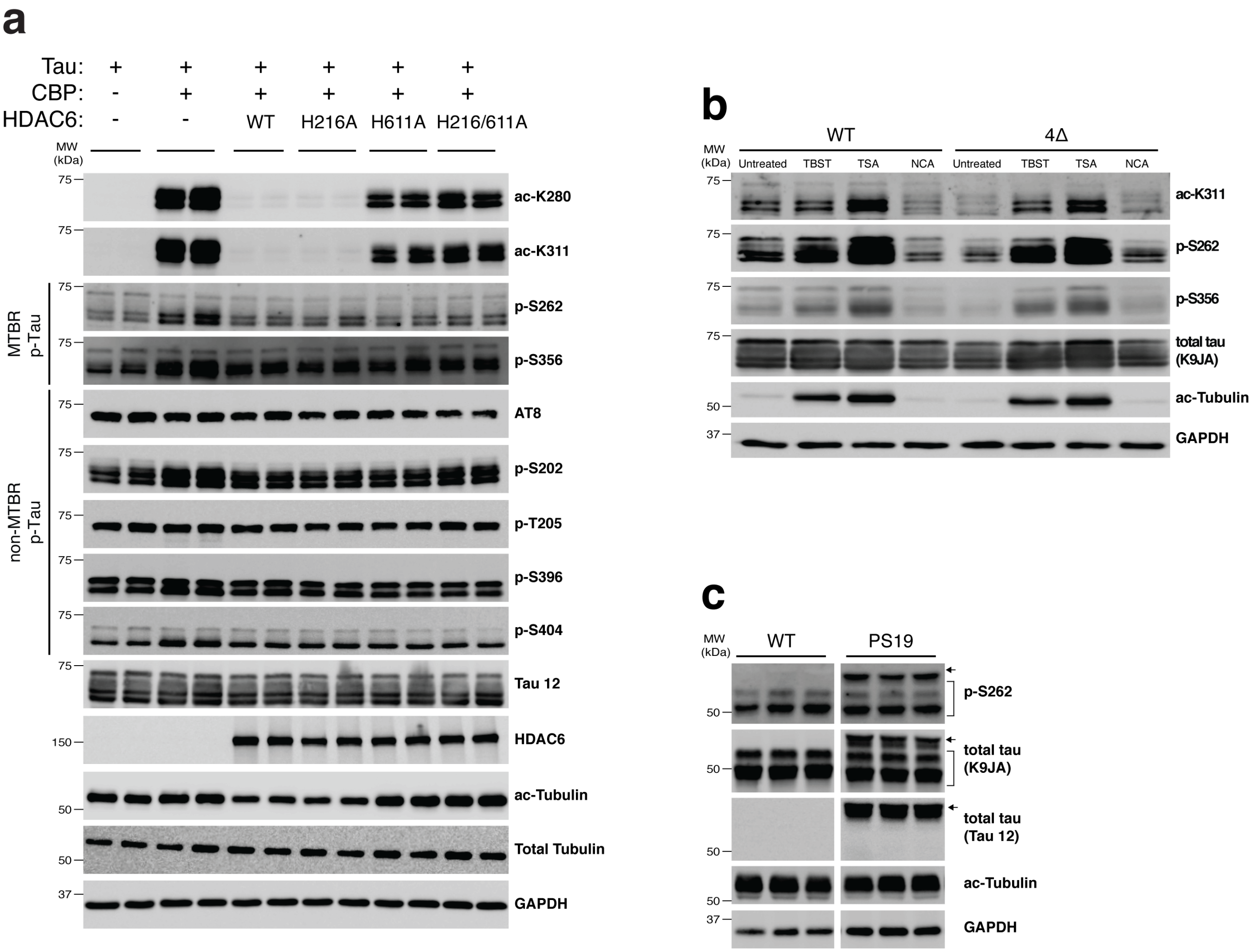

Supplementary Fig. 6 HDAC6 regulates tau acetylation and phosphorylation status**. a** Immunoblotting of lysates from HEK-293A cells transfected with WT tau, the acetyltransferase CBP, and HDAC6 (WT, H216A, H611A, or H216/611A, where indicated) using acetylation or phosphorylation specific tau antibodies. **b** Transfected cells expressing WT tau or 4Δ (Hsc70/Hsp70 binding-deficient) treated with the HDAC inhibitors tubastatin A (TBST, HDAC6 inhibitor, 5 μM), trichostatin A (TSA, class I/II HDAC inhibitor, 1 μM) or nicotinamide (NCA, class III HDAC inhibitor, 5mM) and analyzed by immunoblotting using acetylation or phosphorylation specific tau antibodies. **c** WT and PS19 tau-P301S transgenic neurons derived from mouse embryos (E16) were cultured at 10 DIV and analyzed by immunoblotting. The arrows highlight over-expressed human tau migrating at ~ 65 kDa compared to mouse tau at ~ 50 kDa. WT neurons express only endogenous mouse tau (~ 50 kDa), compared to PS19 neurons that express both human and mouse tau. Representative immunoblotting is shown from n = 4 (a) and n = 3 (b, c) biologically independent experiments. Source data are provided as a Source Data file

Supplementary Fig. 7

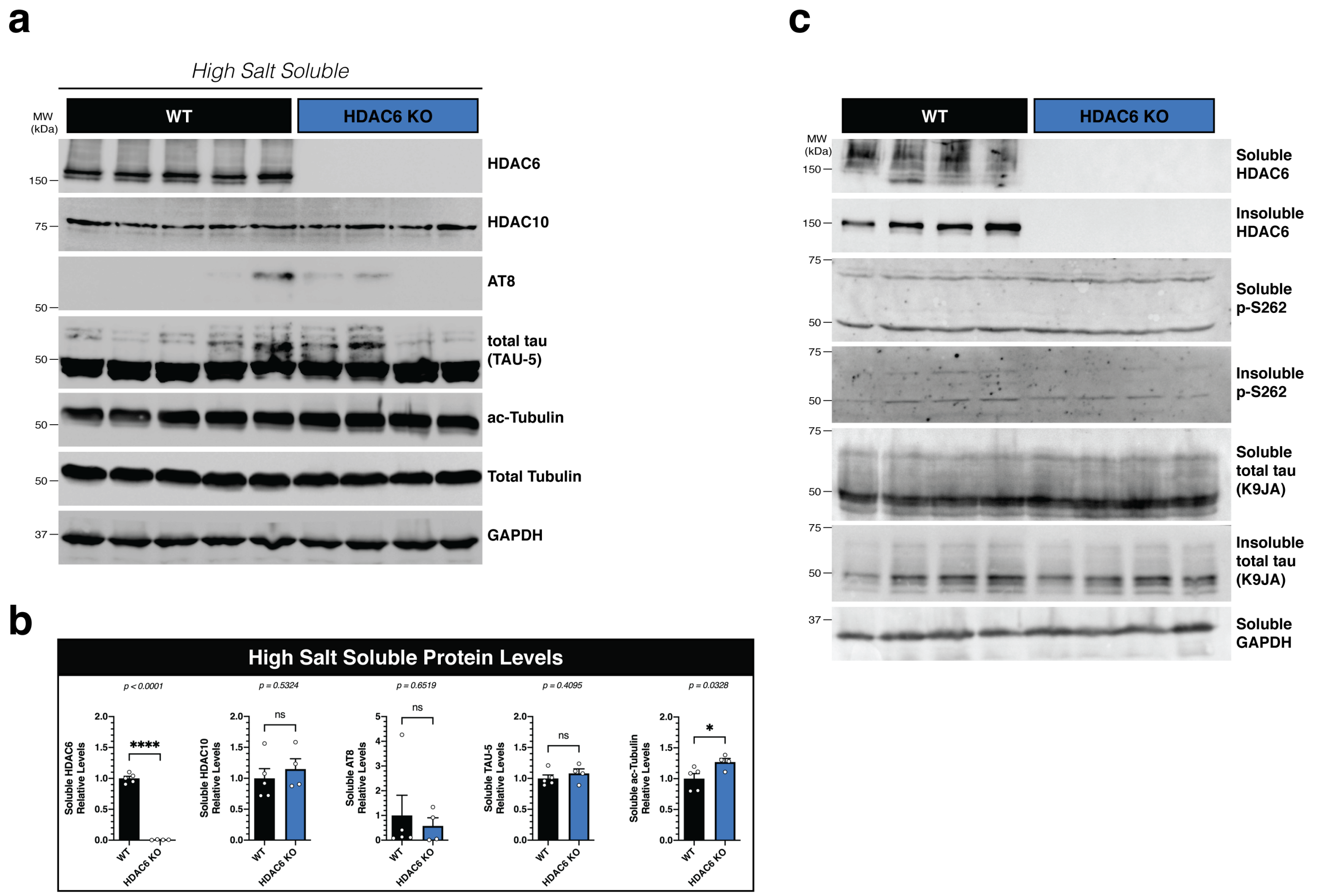

Supplementary Fig. 7 Lack of insoluble tau pathology in HDAC6 KO mice**. a** Immunoblotting of soluble high salt fractions from 12-month old WT (n=5) and HDAC6 KO (n=4) cortical lysates to evaluate the levels of HDAC6, HDAC10 (an unrelated HDAC), phosphorylated tau (AT8), total tau (TAU-5), ac-tubulin, total tubulin, and GAPDH. **b** Immunoblots from **a** were quantified and normalized to WT. **c** Immunoblotting of soluble RIPA-extracted fractions and insoluble SDS-extracted fractions from 12-month old WT (n=4) and HDAC6 KO (n=4) brain lysates to confirm the lack of tau pathology in either fraction using phosphorylated tau (p-S262) and total tau (K9JA) antibodies. Statistical tests: p values determined by two-sided unpaired t-test with Welch's correction for comparisons between WT and HDAC6 KO mice (b). Representative immunoblotting data are shown from n = 4 biologically independent samples (a, c). Error bars represent means ± SEM. *p< 0.05, ****p<0.0001, ns=not significant. Source data are provided as a Source Data file.

Supplementary Fig. 8

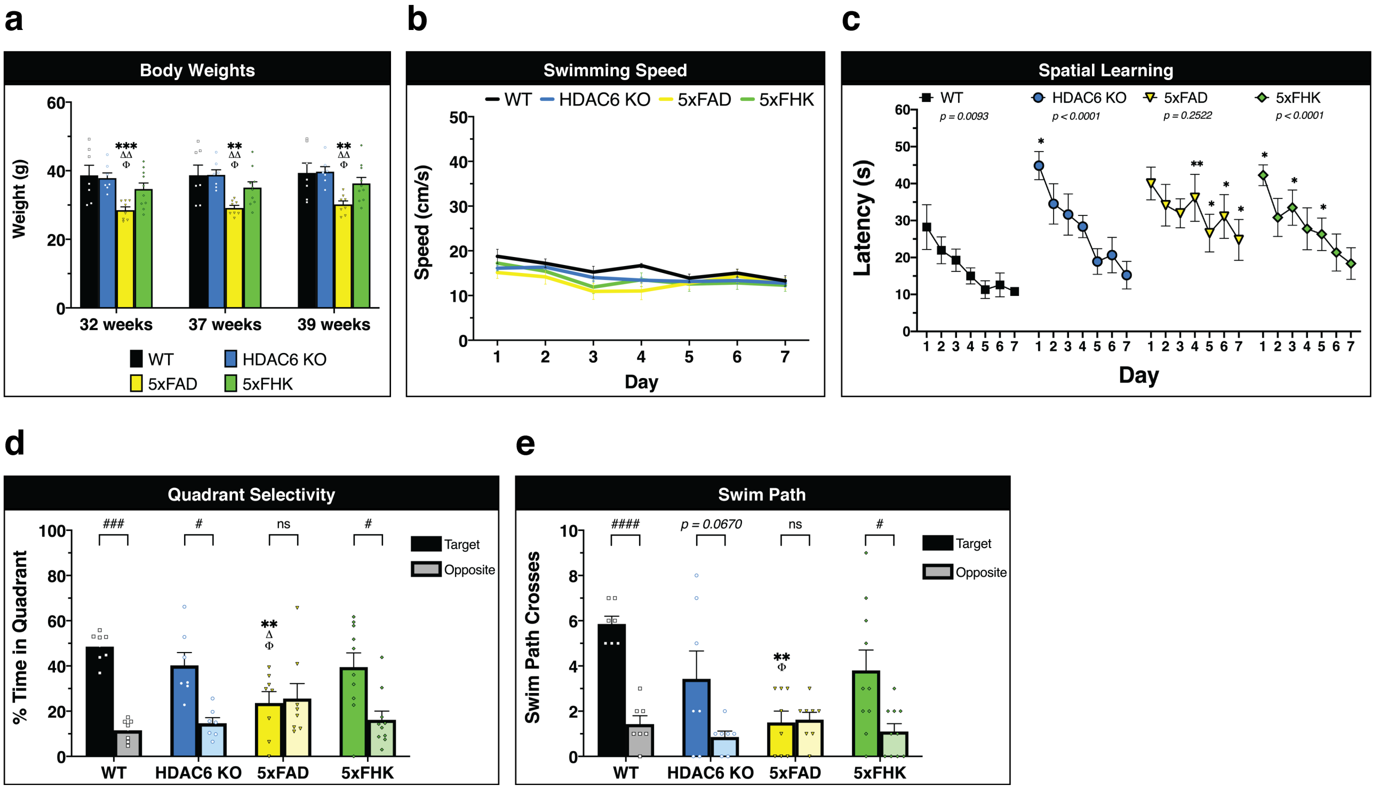

Supplementary Fig. 8 Loss of HDAC6 in the 5xFAD mouse model**.** **a** Decreased body weights were observed in male 5xFAD, but not 5xFHK, mice across the behavior study. Comparisons among genotypes were as follows: **p<0.01, ***p<0.001, comparison to same measure in WT; ΔΔ p<0.01, comparison to HDAC6 KO; ϕ p<0.05, comparison to 5xFHK. **b** Comparable average swimming speeds across genotype during acquisition (training) and reversal learning in the Morris water maze (aged 33-42 weeks). **c** Mice were evaluated for acquisition of spatial learning in a hidden platform task. Data are means ± SEM from four 1-min trials per day. 5xFAD mice showed impaired learning, in comparison to WT. P-values represent within-genotype repeated measures ANOVAs, effect of training day. Asterisks represent comparisons to WT (*p<0.05, **p<0.01, ***p<0.001). **d** Quadrant preference data are means ± SEM from 1-min probe trials without the platform following acquisition learning. Target indicates the quadrant where the platform had been placed during each phase, versus the opposite quadrant. Brackets signify within-genotype comparisons, effect of quadrant (# p<0.05, ### p<0.001, ns = not significant). Between-genotype comparisons: **p<0.01, comparison to WT; Δ p<0.05, comparison to HDAC6 KO, ϕ p<0.05, comparison to 5xFHK. **e** Swim path crosses during 1-min probe trials without the platform following acquisition learning. Target indicates the circular location where the platform had been placed during each phase, versus the corresponding location in the opposite quadrant. Within-genotype comparisons were used to determine effect of quadrant (# p<0.05, ### p<0.001, ns = not significant). Between-genotype comparisons: **p<0.01, comparison to WT and ϕ p<0.05, comparison to 5xFHK. Statistical tests: p value determined by repeated measure ANOVAs followed by Fisher's protected least-significant difference (PLSD) post-hoc tests from WT (n=7), HDAC6 KO (n=7), 5xFAD (n=8), 5xFHK (n=10) independent male mice (a-e). Error bars represent means ± SEM (a-e). Source data are provided as a Source Data file

Supplementary Fig. 9

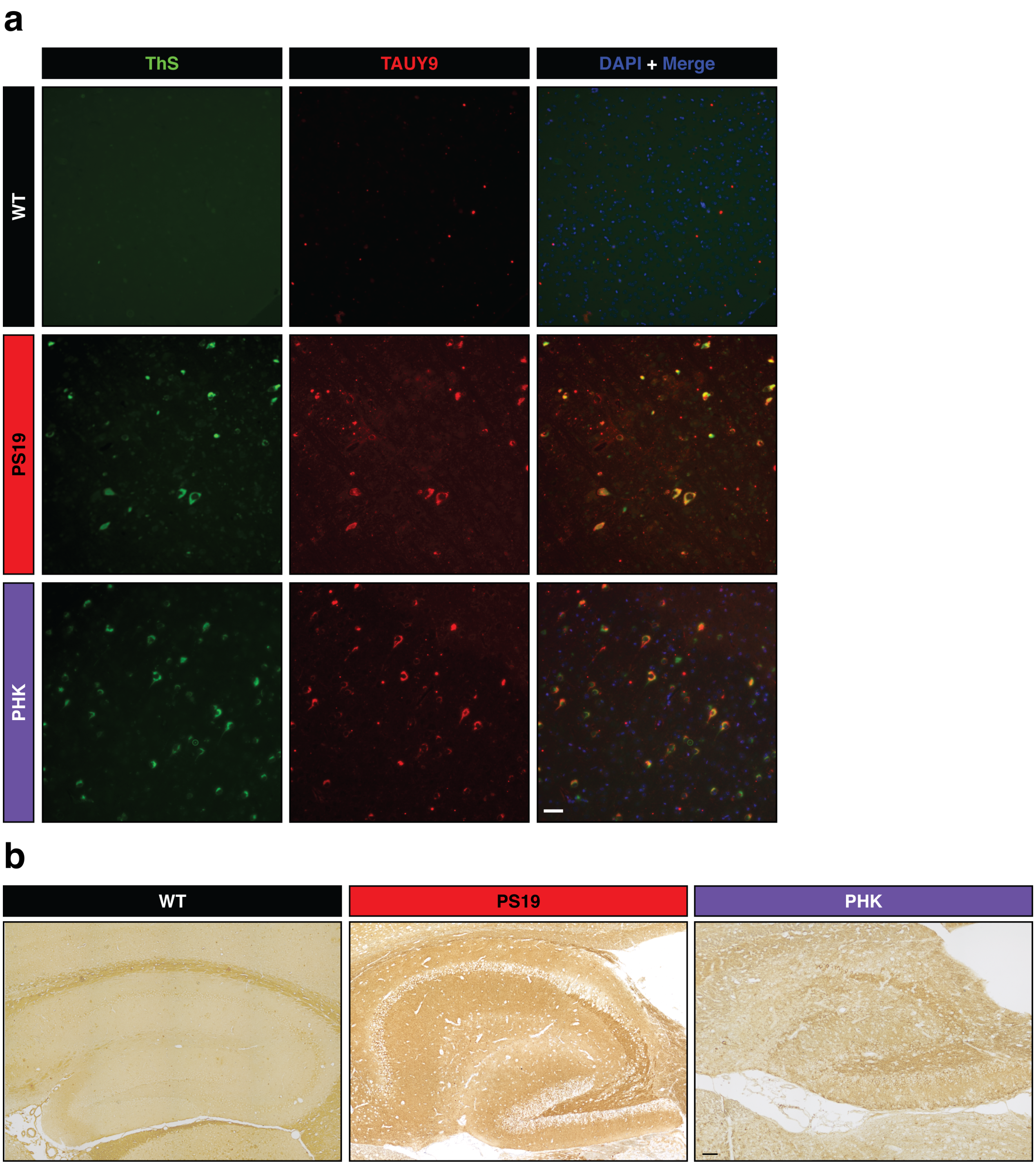

Supplementary Fig. 9 PS19 mice lacking HDAC6 show increased ThS-positive tau inclusions and accelerated hippocampal atrophy**. a** Individual channels are shown for ThS (green), TAUY9 (red), and DAPI + Merge (related to images from Fig. 6d). Scale bar = 50 µm. **b** Representative IHC and immunofluorescence images are depicted from the full hippocampal field from n = 3 biologically independent experiments (a, b) from WT, PS19, and PHK mice, which were used for hippocampal atrophy measurements shown in Fig. 6f. Scale bar = 100 µm.

Supplementary Fig. 10

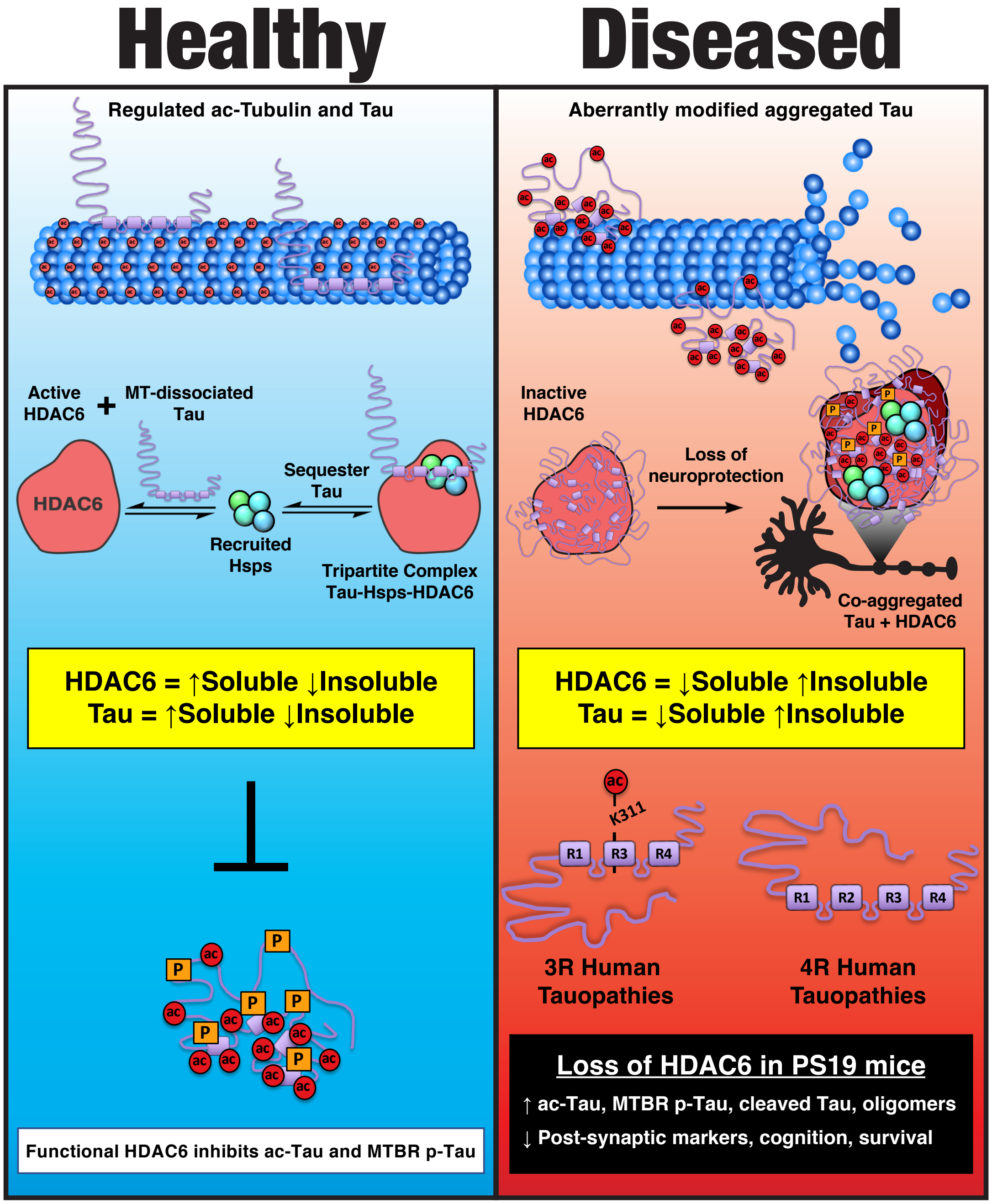

Supplementary Fig. 10 Summary model depicting the role of HDAC6 in healthy (left) and diseased (right) brain.

Supplementary Table 1

Case no.	Diagnosis	Sex	Age at death	Year onset	Duration (years)	ac-K311 positive
1 (IB)	Alzheimer's disease	Female	90	87	3	+
2 (IB)	Alzheimer's disease	Male	66	58	8	+
3 (IB)	Alzheimer's disease	Female	83	77	6	+
4 (IB)	Alzheimer's disease	Female	56	47	9	+
5 (IB)	Alzheimer's disease	Female	61	53	8	+
6 (IHC, IF)	Alzheimer's disease	Male	74	N/A	N/A	+
7 (IHC)	Alzheimer's disease	Female	76	68	8	+
8 (IHC)	Alzheimer's disease	Male	60	56	4	+
9 (IHC)	Alzheimer's disease	Male	86	80	6	+
10 (IHC)	Alzheimer's disease	Female	87	69	18	+
11 (IHC)	Alzheimer's disease	Female	80	68	12	+
12 (IHC)	Alzheimer's disease	Male	69	58	11	+
13 (IHC)	Alzheimer's disease	Female	72	62	10	+
14 (IHC)	Alzheimer's disease	Female	80	N/A	N/A	+
15 (IHC)	Clinical history of schizophrenia	Male	83	N/A	N/A	+
16 (IHC, IF)	Corticobasal degeneration	Male	86	80	6	-
17 (IHC)	Corticobasal degeneration	Female	56	50	6	-
18 (IHC)	Corticobasal degeneration	Male	56	50	6	-
19 (IHC)	Corticobasal degeneration	Male	66	60	6	-
20 (IHC)	Corticobasal degeneration	Male	60	55	5	-
21 (IHC)	Corticobasal degeneration	Male	63	59	4	-
22 (IHC)	Corticobasal degeneration	Female	71	65	6	-
23 (IHC)	Corticobasal degeneration	Female	79	73	6	-
24 (IHC)	Corticobasal degeneration	Male	71	69	2	-
25 (IHC)	Corticobasal degeneration	Female	61	54	7	-
26 (IHC)	Corticobasal degeneration	Female	70	66	4	-
27 (IHC)	Corticobasal degeneration	Male	74	67	7	-
28 (IHC)	Corticobasal degeneration	Male	75	71	4	+
29 (IHC)	Corticobasal degeneration	Female	64	56	8	-
30 (IHC)	Corticobasal degeneration	Male	73	72	1	-
31 (IHC)	Fronototemporal lobar degeneration (MAPT P301L Mutation)	Female	65	56	9	-
32 (IHC)	Parkinson's disease, atypical	Female	77	73	4	-
33 (IHC)	Pathological Aging	Female	65	N/A	N/A	-
34 (IHC, IF)	Pick's disease	Male	71	58	13	+
35 (IHC)	Pick's disease	Male	76	69	7	+
36 (IHC)	Pick's disease	Male	72	57	15	+
37 (IHC)	Primary age-related tauopathy	Female	83	N/A	N/A	+
38 (IHC, IF)	Progressive supranuclear palsy	Male	92	89	3	-
39 (IHC)	Progressive supranuclear palsy	Female	71	67	4	-
40 (IHC)	Progressive supranuclear palsy	Female	76	73	3	-
41 (IHC)	Progressive supranuclear palsy	Male	73	69	4	-
42 (IHC)	Progressive supranuclear palsy	Male	72	62	10	-
43 (IHC)	Progressive supranuclear palsy	Male	66	62	4	-
44 (IHC)	Progressive supranuclear palsy	Male	71	64	7	-
45 (IHC)	Progressive supranuclear palsy	Female	78	67	11	-
46 (IHC)	Progressive supranuclear palsy	Male	48	46	2	-
47 (IHC)	Progressive supranuclear palsy	Male	80	75	5	-
48 (IHC)	Progressive supranuclear palsy	Male	72	63	9	-
49 (IHC)	Progressive supranuclear palsy	Male	79	73	6	-
50 (IHC)	Progressive supranuclear palsy	Male	81	33	48	-
51 (IHC)	Progressive supranuclear palsy	Female	71	65	6	-
52 (IHC)	Progressive supranuclear palsy	Female	79	70	9	-
53 (IHC)	Progressive supranuclear palsy	Male	69	66	3	-
54 (IHC)	Progressive supranuclear palsy	Female	72	69	3	-
55 (IHC)	Progressive supranuclear palsy	Female	63	58	5	+
56 (IHC)	Progressive supranuclear palsy	Female	82	79	3	-
57 (IHC)	Progressive supranuclear palsy	Male	72	67	5	+
58 (IHC)	Progressive supranuclear palsy	Male	79	70	9	-
59 (IHC)	Progressive supranuclear palsy	Female	71	61	10	-
60 (IHC)	Progressive supranuclear palsy	Male	84	76	8	+
61 (IHC)	Progressive supranuclear palsy	Male	71	64	7	-
62 (IHC)	Progressive supranuclear palsy	Male	81	72	9	-
63 (IHC)	Progressive supranuclear palsy	Male	75	67	8	-
64 (IHC)	Progressive supranuclear palsy	Female	78	74	4	-
65 (IHC)	Tauopathy unclassifiable	Male	63	52	11	-
66 (IB)	Unremarkable adult brain	Female	92	N/A	N/A	-
67 (IB)	Unremarkable adult brain	Female	83	N/A	N/A	-
68 (IB)	Unremarkable adult brain	Male	73	N/A	N/A	-
69 (IHC)	Unremarkable adult brain	Male	62	N/A	N/A	-
70 (IHC)	Unremarkable adult brain	Male	66	N/A	N/A	-

Supplementary Table 1 List of human tauopathy cases and demographics from this study. Demographics for all human subjects are listed. Cases were used for immunoblotting (IB), immunofluorescent (IF), and immunohistochemistry (IHC) analyses. Red denotes cases that exhibited positive immunoreactivity for ac-K311 pathology (last column). The extent of ac-K311 immunoreactivity was determined based on a semi-quantitative scoring measure[1].

Supplementary Table 2

Antibody	Dilution	Source	Catalog #
Primary:			
**ac-K280**	1:250 (IB)	Cohen, et al., 2011	
**ac-K311 #5089**	1:250 (IB)<br>1:500 (IF)<br>1:1000 (IHC)	From this study	
**ac-K311 #5090**	1:250 (IB)	From this study	
**ac-K311 #5091**	1:250 (IB)	From this study	
**ac-K311 #5092**	1:250 (IB)	From this study	
**ac-Tubulin**	1:1000 (IB)	Sigma-Aldrich	T7451
**AT8**	1:500 (IB)<br>1:1000 (IHC)	ThermoFisher	MN1020
**FLAG (FL)**	1:1000 (IB, IF)	Sigma-Aldrich	F1804
**GAPDH**	1:1000 (IB)	Millipore	ABS16
**GFP**	1:1000 (IB, IF)	Santa Cruz	sc-9996
**HDAC6 (H&M)**	1:500 (IB)	Millipore	07-732
**HDAC6 (H)**	1:200 (IF)<br>1:1000 (IB)	Santa Cruz	sc-28386
**HDAC6 (H&M)**	1:200 (IF)<br>1:250 (IB)	Generous gift from Tso-Pang Yao	
**HDAC10 (H&M)**	1:250 (IB)	Generous gift from Tso-Pang Yao	
**Hsc70**	1:1000 (IB)	Enzo	ADI-SPA-815
**Hsp70**	1:1000 (IB)	Enzo	ADI-SPA-810
**K9JA (H&M)**	1:5000 (IB)	DAKO	A0024
**MC1**	1:200 (IF)<br>1:1000 (IHC)	Generous gift from Peter Davies	
**Myc**	1:1000 (IB)	Santa Cruz	sc-40
**p-NR2B**	1:500 (IB)	PhosphoSolutions	p1516-1472
**p-S202**	1:1000 (IB)	Abcam	ab108387
**p-S262**	1:1000 (IB)	ThermoFisher	44-750G
**p-S356**	1:1000 (IB)	ThermoFisher	44-751G
**p-S396**	1:1000 (IB)	ThermoFisher	44-752G
**p-S404**	1:1000 (IB)	ThermoFisher	44-758G
**p-T205**	1:1000 (IB)	Abcam	ab4841
**PSD-95**	1:500 (IB)	Abcam	ab18258
**RD3**	1:250 (IB)<br>1:500 (IF)	Millipore	05-803
**RD4**	1:250 (IB)<br>1:5000 (IF)	Millipore	05-804
**Synaptophysin**	1:1000 (IB)	Millipore	MAB5258
**T14**	N/A (IP, MS)	ThermoFisher	13-1400
**T46**	N/A (IP, MS)	ThermoFisher	13-6400
**Tau 12 (H)**	1:1000 (IB, IHC)	Millipore	MAB2241
**Tau-1**	1:1000 (IB)<br>1:5000 (IF)	Millipore	MAB3420
**TAU-5 (H&M)**	1:500 (IF)<br>1:1000 (IB, IHC)	ThermoFisher	AHB0042
**TauC3**	1:250 (IB)	ThermoFisher	AHB0061
**TAUY9 (H)**	1:500 (IF)	Enzo	BML-TA3119-0100
**TOC1**	1:500 (DB)	Generous gift from Nicholas Kanaan	
**Total NR2B**	1:1000 (IB)	Millipore	06-600
**Total Tubulin**	1:1000 (IB)	Sigma-Aldrich	T6199

Antibody	Dilution	Source	Catalog #
Secondary:			
**Donkey anti-Mouse IgG (H+L) Highly Cross-Adsorbed Secondary Antibody, Alexa Fluor 647**	1:500 (IF)	ThermoFisher	A-31571
**Donkey anti-Rabbit IgG (H+L) Highly Cross-Adsorbed Secondary Antibody, Alexa Fluor 568**	1:500 (IF)	ThermoFisher	A10042
**Goat anti-Mouse IgG Antibody (H+L), Biotinylated**	1:1000 (IHC)	Vector Laboratories	BA-9200
**Goat anti-Rabbit IgG Antibody (H+L), Biotinylated**	1:1000 (IHC)	Vector Laboratories	BA-1000
**Goat anti-Mouse IgG (H+L) Cross-Adsorbed Secondary Antibody, Alexa Fluor 488**	1:500 - 1:1000 (IF)	ThermoFisher	A-11001
**Goat anti-Rabbit IgG (H+L) Cross-Adsorbed Secondary Antibody, Alexa Fluor 594**	1:500 (IF)	ThermoFisher	A-11012
**Horse anti-Mouse IgG (H+L), Biotinylated**	1:1000 (IHC)	Vector Laboratories	BA-2000

Supplementary Table 2 List of sources and catalog numbers for all primary and secondary antibodies used in this study. Shown are antibodies and dilutions used for immunoprecipitation assays (IP), mass spectrometry (MS), dot blotting (DB), immunoblotting (IB), immunofluorescence microscopy (IF), and immunohistochemistry (IHC). Human and mouse antibody species reactivity is denoted by (H) and (M), respectively.

Supplementary References

1. Irwin DJ, Cohen TJ, Grossman M, Arnold SE, Xie SX, Lee VM, Trojanowski JQ. Acetylated tau, a novel pathological signature in Alzheimer's disease and other tauopathies. *Brain* **135**, 807-818 (2012).
