# Supplementary material for: An HDAC6-dependent surveillance mechanism suppresses tau-mediated neurodegeneration and cognitive decline
Source: Nat Commun. 2020 Nov 2;11:5522. doi: 10.1038/s41467-020-19317-4 (PMC7606452; doi:10.1038/s41467-020-19317-4)
Supplement: Supplementary file 3 — Reporting Summary [file 41467_2020_19317_MOESM3_ESM.pdf]

nature research

Corresponding author(s): Todd J Cohen

Last updated by author(s): Sep 27, 2020

Reporting Summary

Nature Research wishes to improve the reproducibility of the work that we publish. This form provides structure for consistency and transparency in reporting. For further information on Nature Research policies, see Authors & Referees and the Editorial Policy Checklist .

Statistics

For all statistical analyses, confirm that the following items are present in the figure legend, table legend, main text, or Methods section.

n/a	Confirmed	
☐	☒	The exact sample size (*n*) for each experimental group/condition, given as a discrete number and unit of measurement
☐	☒	A statement on whether measurements were taken from distinct samples or whether the same sample was measured repeatedly
☐	☒	The statistical test(s) used AND whether they are one- or two-sided<br>*Only common tests should be described solely by name; describe more complex techniques in the Methods section.*
☐	☒	A description of all covariates tested
☐	☒	A description of any assumptions or corrections, such as tests of normality and adjustment for multiple comparisons
☐	☒	A full description of the statistical parameters including central tendency (e.g. means) or other basic estimates (e.g. regression coefficient) AND variation (e.g. standard deviation) or associated estimates of uncertainty (e.g. confidence intervals)
☐	☒	For null hypothesis testing, the test statistic (e.g. *F*, *t*, *r*) with confidence intervals, effect sizes, degrees of freedom and *P* value noted<br>*Give P values as exact values whenever suitable.*
☒	☐	For Bayesian analysis, information on the choice of priors and Markov chain Monte Carlo settings
☒	☐	For hierarchical and complex designs, identification of the appropriate level for tests and full reporting of outcomes
☒	☐	Estimates of effect sizes (e.g. Cohen's *d*, Pearson's *r*), indicating how they were calculated

Our web collection on statistics for biologists contains articles on many of the points above.

Software and code

Policy information about availability of computer code

Data collection	Xcalibur software (Thermo Scientific), Nikon NIS Elements software, Omega Reader Control software, DYNAMICS 7.1.7.16 Software, Noldus Ethovision, ImageQuant TL v8.1
Data analysis	Adobe Photoshop, version 9.0, and MARS Data Analysis Software, ImageJ bundled with Java 1.8.0_172, Mascot, Scaffold, and PEAKS 6.0 (Bioinformatics Solutions Inc.), ImageQuant TL Analysis Toolbox

For manuscripts utilizing custom algorithms or software that are central to the research but not yet described in published literature, software must be made available to editors/reviewers. We strongly encourage code deposition in a community repository (e.g. GitHub). See the Nature Research guidelines for submitting code & software for further information.

Data

Policy information about availability of data

All manuscripts must include a data availability statement. This statement should provide the following information, where applicable:

- Accession codes, unique identifiers, or web links for publicly available datasets
- A list of figures that have associated raw data
- A description of any restrictions on data availability

The authors declare that all data supporting the findings of this study are available within the paper and its supplementary information files.

Field-specific reporting

Please select the one below that is the best fit for your research. If you are not sure, read the appropriate sections before making your selection.

☒ Life sciences ☐ Behavioural & social sciences ☐ Ecological, evolutionary & environmental sciences

Life sciences study design

All studies must disclose on these points even when the disclosure is negative.

Sample size	Sample sizes were determined to provide maximum reliability and biological significance using the minimum number of mice/samples. Sample numbers for all animal studies were predetermined as follows. The number of mice proposed is based on power calculations (minimum 80% power) using the minimum number of mice. The sample sizes chosen for survival allows us to detect an 8% difference in survival rate, which equates to +/- 1 month differences in survival in the PS19 model. n > 20 animals per genotype were used for survival, n > 8 animals per genotype for behavior analysis, and n > 4 animals per genotype for all histology and biochemistry experiments. All cell and neuron based experiments throughout this study employed n > 3 biological replicates with appropriate reproducibility to provide adequate statistical analysis.
Data exclusions	no data were excluded
Replication	only experiments that were replicated are described in this study. A minimum of n=3 independent biological replicates and n=3 technical replicates were used throughout. All human tissue analysis were replicated by biochemistry and histological assays. (n>5 brain samples). In no instance are data shown that have not been replicated.
Randomization	Samples were allocated based on genotype, treatment condition, disease phenotype, age, or sex. Only one variable was examined under each experimental comparison. All comparisons in animals were normalized to age and sex. Animal behavior was performed in mixed sex cohorts but reported in this study for males only. To account for confounding experimental variation in immunoblotting and staining, samples were re-analyzed using freshly made solutions and gels and placed in varying physical positions during the analysis to account for placement or spatial effects.
Blinding	All survival and behavior analysis were performed by personnel that were blinded to animal genotypes. The quantification of immunoblotting, histology, immunofluorescence, and immunohistochemistry data was performed by three independent lab personnel that were presented with data sets containing randomly coded/labeled images.

Reporting for specific materials, systems and methods

We require information from authors about some types of materials, experimental systems and methods used in many studies. Here, indicate whether each material, system or method listed is relevant to your study. If you are not sure if a list item applies to your research, read the appropriate section before selecting a response.

Materials & experimental systems

n/a	Involved in the study	
☐	☒	Antibodies
☐	☒	Eukaryotic cell lines
☒	☐	Palaeontology
☐	☒	Animals and other organisms
☐	☒	Human research participants
☒	☐	Clinical data

Methods

n/a	Involved in the study	
☒	☐	ChIP-seq
☒	☐	Flow cytometry
☒	☐	MRI-based neuroimaging

Antibodies

Antibodies used	All antibodies used in this study are now listed in Supplementary Table 2. All secondary antibodies were used at 1:500 or 1:1000 dilution, which has now been included in the methods section. Commercial sources are listed in the table as well, and any gifted antibodies are acknowledged in the methods or acknowledgments section.
Validation	All tau and acetylated tau antibodies used in this study (ac-K280 and ac-K311) were validated 1) using tau knockout mice to show loss of tau signal, 2) using over-expression of tau plasmids in the presence or absence of the acetyltransferase CBP in HEK-293A cells as positive controls, 3) using omission of primary antibody to test non-specific background binding (but the presence of secondary anti-IgG antibodies), 4) using K280R and K311R mutant tau plasmids (in which the acetylated lysine residue is mutated to arginine) to demonstrate abolished ac-K280 and ac-K311 antibody immunoreactivity.

Eukaryotic cell lines

Policy information about cell lines

Cell line source(s)	HEK-293A cells are provided by ThermoFisher, #R70507
Authentication	The commercial HEK-293A cells used were not authenticated

Mycoplasma contamination	All cell lines tested negative for mycoplasma contamination
Commonly misidentified lines (See ICLAC register)	none

Animals and other organisms

Policy information about studies involving animals; ARRIVE guidelines recommended for reporting animal research

Laboratory animals	Wild-type, HDAC6 KO, PS19, and 5xFAD strains (C57BL/6 background) aged 6-12 months old were used throughout this study for behavior analysis. Equal male/female ratios were used in all instances for behavior, histology, biochemistry, and survival data. While data from both sexes was acquired for behavior, it was reported for only males in the main and supplementary figures. For embryonic neurons, 8 week old female CD1 or C57BL/6 mice were used for timed pregnant breeding and cortical neurons were dissected from E16 embryos. Breeding cages for general maintenance and culling always employs equal ratios of males and females. Housing conditions: a 14-hour light/10-hour dark cycle is used, temperature range is from 65-75°F with 40-60% humidity.
Wild animals	none
Field-collected samples	this study did not involve field-collected samples
Ethics oversight	All protocols were carried out in accordance with the University of North Carolina (UNC) Institutional Animal Care and Use Committee (UNC IACUC protocol 19.017).

Note that full information on the approval of the study protocol must also be provided in the manuscript.

Human research participants

Policy information about studies involving human research participants

Population characteristics	*Describe the covariate-relevant population characteristics of the human research participants (e.g. age, gender, genotypic information, past and current diagnosis and treatment categories). If you filled out the behavioural & social sciences study design questions and have nothing to add here, write "See above."*
Recruitment	*Describe how participants were recruited. Outline any potential self-selection bias or other biases that may be present and how these are likely to impact results.*
Ethics oversight	For human studies, fixed, paraffin-embedded tissue blocks or frozen tissue were obtained from the Center for Neurodegenerative Disease Research Brain Bank at the University of Pennsylvania. Consent for autopsy was obtained from legal representatives for all subjects in accordance with local institutional review board requirements at University of Pennsylvania.

Note that full information on the approval of the study protocol must also be provided in the manuscript.
